# The Prevalence of Cardiovascular Diseases in Paralympic Athletes

**DOI:** 10.3390/healthcare11071027

**Published:** 2023-04-04

**Authors:** Diana Sawczuk, Paweł Gać, Rafał Poręba, Małgorzata Poręba

**Affiliations:** 1 Department of Internal Medicine, Occupational Diseases, Hypertension and Clinical Oncology, Wroclaw Medical University, Borowska 213, 50-556 Wroclaw, Poland; 2 Department of Population Health, Division of Environmental Health and Occupational Medicine, Wroclaw Medical University, Mikulicza-Radeckiego 7, 50-368 Wroclaw, Poland; 3 Department of Paralympic Sports, Wroclaw University of Health and Sport Sciences, Witelona 25a, 51-617 Wroclaw, Poland

**Keywords:** arrhythmia, cardiovascular disease, electrocardiography, paralympic athletes

## Abstract

Paralympic participants represent a special subset of athletes. Although sudden cardiac death in this group is a rare event, it should be underlined that, in particular, Paralympians with movement restrictions have a higher prevalence of coronary heart disease. Numerous reports have focused on comparing athletes with spinal cord injury (SCI) and the ones with non-spinal cord injury—NSCI. The first group is more prone to develop arrhythmias, arterial hypertension, hyperlipidaemia including atrial fibrillation and atrial flutter, and this group potentially may have a higher risk of cardiovascular mortality. In ECGs of the disabled athletes with SCI, we more often find changes typically established as consequences of exercise training, such as T-wave inversions. The potential differences in the cardiovascular status of disabled athletes may depend not only on the class of impairment, but also on the discipline of sport and environmental conditions, which makes the analysis relatively complex. The paper analyses up-to-date articles discussing the cardiovascular problems in disabled athletes, pointing to scarce data in several fields of interest. Previous studies on the frequency of abnormalities of the cardiovascular system in Paralympic athletes highlighted the need to intensify preventive cardiology care for this group of athletes, and some activities could be proposed for sportsmen and sportswomen in this group, including more frequent screening ECG, application of 24 h ECG Holter monitoring, echocardiography and cardiological care. Due to the relatively few data available and existing discrepancies in this area, further research is necessary.

## 1. Introduction

For more than 100 years, athletes with various disabilities have been able to participate in the official sports events. Since 1960, when the first Paralympic Games took place in Rome, we have paid increasing attention to individuals with physical and mental impairments [1]. Olympic and Paralympic participants represent a special subset of athletes that became a model of success all over the world by enduring strenuous exercise and achieving astonishing performances [2].

Sport training and performance should be considered in light of the fundamental changes in physiological adjustment [1]. Despite their astounding achievements, athletes are not immune to cardiovascular diseases. This is why every sports physician must consider the increased risk of injuries and illness in elite athletes, especially among individuals with impairments, and try to find the balance between health management and enhanced sport performance.

Although the health benefits of physical activity are clear, it should be underlined that any exercise can trigger sudden cardiac arrest, especially in people with cardiovascular abnormalities [3]. Sudden cardiac death in athletes is a rare event; nevertheless, according to the increasing number of people practising high-performance sports, it became a matter of significant consideration [4]. This issue seems to be particularly important in the group of athletes with SCI, especially with the movement restrictions, as there were reports indicating the increased incidence of coronary artery disease among patients with this disability, and it is known that the coronary disease is an important cause of mortality worldwide [5]. One study showed that approximately 34% of patients with spinal cord injuries suffer from hypertension, ischemic heart disease or diabetes mellitus compared to 18.6% of the control group [5]. The increased risk of cardiovascular diseases is probably attributable to a higher prevalence of structural cardiac changes due to haemodynamic alternations in those with spinal cord injuries and in amputees [6].

The paralympic sports are becoming more and more popular, and this has created new challenges for the physicians that work with impairments. For this reason, a newly adopted term—“paralympic medicine”—was created to describe the varied healthcare issues associated with Paralympic athletes [7].

## 2. Materials and Methods

**Figure 1:**
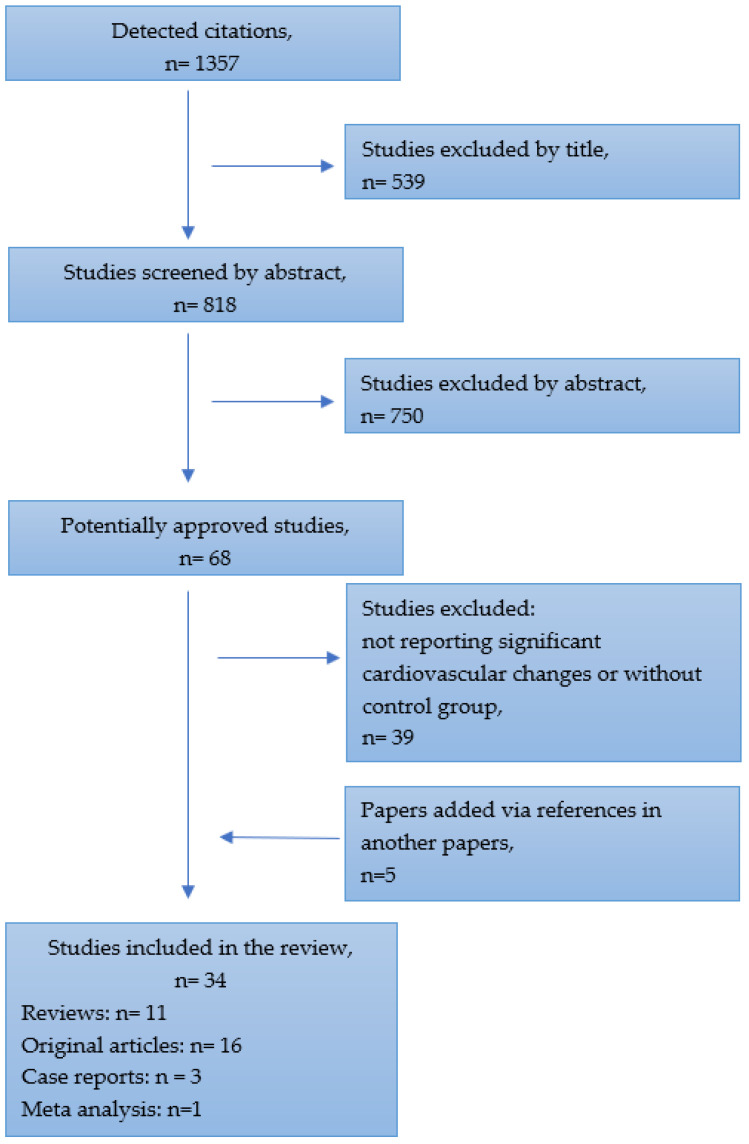
Flow chart for selecting studies.

The literature analysis was performed using PubMed, Google Scholar and Directory of Open Access Journals databases, from 1985 to November 2022. All types of articles were included, such as reviews, case reports, meta-analyses, original articles and cohort trials. A search was conducted to source published information for all available studies reporting Paralympic athletes’ health issues compared with those of able-bodies athletes. From the initial detected citations, the review included studies that incorporate cardiovascular problems which may be related to certain kinds of disabilities and sports disciplines. The details of the selection process are presented in Figure 1.

The following keywords were used: cardiovascular diseases, athlete, Olympics, Paralympics, disabled, paralympic sports, arrhythmias, atrial fibrillation, atrial flutter, sudden cardiac death.

## 3. Results

### 3.1. General Characteristics of the Paralympian’s Cardiovascular System

According to Pelliccia et al., cardiac remodelling in Paralympic athletes is differentiated by disability and sports discipline [8].

It is essential to know the phenotypic features that predispose athletes to a better physical performance and more health benefits. There is still large unexplained variability in exercise-induced cardiac remodelling among highly trained endurance athletes, and especially among the disabled athletes [9]. For this reason, for the first time, the multicentre prospective cohort trial was planned to identify the impact of training load and genotype on the variance of exercise-induced cardiac remodelling [9]. The study underlines how essential is to determine the association of sport-induced cardiac remodelling with physical performance, health benefits and cardiac pathology. Furthermore, the long-term cohort study would include the training load, as well as the rare variants in cardiomyopathy-associated genes and polygenic risk scores for cardiovascular features. This type of large-scale research has not been conducted so far for the cohort of disabled athletes.

Paralympic athletes’ various impairments can lead to specific limitations and health issues related to the cardiorespiratory, metabolic and thermoregulatory system [10]. During Para alpine and snow-sport events, athletes have to face altitude acclimatisation, cold conditions, travel fatigue and jetlag [10]. Considering altitude challenges, data have shown that athletes with a spinal cord injury (SCI) report a high rate of acute mountain sickness and diminished ability to adjust to altered barometric pressure [1,10]. Other studies have shown that the magnitude of thermoregulatory limitation in the group of SCI is proportional to the level of injury. Disruption of the autonomic nervous system leads to an absence of or delay in vasoconstriction, which is why athletes are susceptible to hypothermia [11] (Handrakis, Trbovich, Hagen et al., 2017). According to Griggs et al., sportsmen with a limb deficiency can potentially lose heat more rapidly due the reduction in their skeletal mass and greater metabolic heat production as a consequence of movement asymmetries [12] (Griggs, Stephenson, Price i Goosey-Tolfrey, 2019).

On the other hand, due to the aforementioned reduced thermoregulatory ability and hot and/or humid climate conditions, Paralympic athletes competing in Summer Paralympic Games may be at increased risk of exertional heat illness (EHI) or heat-stress related symptoms such as collapse, vomiting, headache, cramping, nausea or dizziness [13]. According to an online survey administered by Alkemade et al. to a subset of 107 Paralympic athletes participating in the Tokyo 2020 Paralympic Games, more than half of participants had experienced heat-stress related symptoms in the past and 21% of them suffered from these symptoms in Tokyo [13].

All Paralympic athletes should undergo evaluation, regardless of age, sex and an associated disability. It should be the role of the sport medicine physicians, and especially team physicians, not only to provide medical care for athletes, but also to participate in physiological monitoring and analyse the risk factors, including the climatic conditions of the place in which the Paralympic Games are held. When considering a cold climate, it is recommended to:

- Prepare a plan for prolonging period of altitude acclimatisation;
- Monitor the load during training, competitions and daily life;
- Carefully monitor the competitors for hypothermia and deal with signs of it earlier in athletes with SCI than in able-bodied athletes [10].

For heat-vulnerable Paralympic sportsmen, strategies of implementation of cooling methods and heat acclimation should be employed, considering the characteristics of the sports discipline and impairment [12].

To safeguard the integrity of fair competition, the official paralympic classification is based on the type of impairment that affects athletes’ ability to perform in different sports. The groupings of athletes by the degree of limitation are called “Sport Classes” and these data are presented in Tables S1–S4 (the data available in the Supplementary Materials).

In studies performed by Pelliccia et al., the physiological and clinical features depended on the type of disability. The athletes were thus divided into two groups: those with spinal cord injury (SCI) and those with non-spinal cord injury—NSCI [8]. Additionally, the authors adopted numerous athletes from variety of disciplines, classified in two main groups:

(1) Endurance sports, including rowing, swimming, cycling, hand-bicycle, long-distance and marathon running, and cross-country skiing;

(2) Non-endurance sports, including (most commonly) fencing, basketball, alpine skiing, ice-sledge hockey, table tennis, and archery, which has been one of the most profound research topics in this area.

One of the major differences was shown in the echocardiographic examination—the SCI male athletes had significantly smaller left ventricular (LV) end-diastolic dimension, as well as LV mass index values, in comparison with NSCI athletes (*p* < 0.001). With reference to type of sports, endurance sports male athletes had statistically significant larger LV cavities (median 53.5 mm vs. 52.0 mm) (with *p* < 0.001), septal left ventricle thickness (median 10 mm vs. 9 mm) and greater LV mass index (median 109 g/m^2^ vs. 88.4 g/m^2^) in contrast with athletes in non-endurance sports (*p* < 0.001) [8].

The consequences of an SCI are numerous and might involve impaired voluntary muscle function, deficits in deep and superficial sensitivity, autonomic dysreflexia and thermal dysregulation [1,14]. Moreover, persons with spinal cord injuries have impaired the descending spinal sympathetic pathways, providing tonic control to preganglionic neurons that are involved in heart race control [15]. This impairment leads to increased risk of cardiovascular diseases, sudden cardiac death, lethal arrhythmias and cardiovascular mortality [16,17,18]. The severity varies and is determined by the level and completeness of the spinal lesion. For example, due to circulatory limitations in the paralysed tissues, paraplegic people experience reduced exercise capacity and increased heart rate responses compared to non-disabled people [19]. Moreover, athletes with cervical or dorsal lesions down to Th6 have limited maximal heart rates owing to a lack of sympathetic drive to the heart. Blood redistribution from body areas lacking autonomic control is impaired, thus reducing venous return [1].

Studies performed by Squair et al. [20] on a group of twenty-six athletes with cervical SCI shows novel evidence that autonomic tests can correlate with the in-competition cardiovascular response. A few autonomic tests were used, including sympathetic skin response, baseline hemodynamics, orthostatic challenge test and cold-pressor tests. One of them, the orthostatic test, predicted approximately 50% of the in-competition peak heart rate (*p* < 0.001). The research can be used to determine the cardiovascular capacity of SCI individuals during a sports performance.

Persons with spinal cord injuries have been characterised as extremely sedentary, with increased risk of secondary complications such as hypertension, atherogenic lipid profile and diabetes mellitus [19]. Because of immobilisation, we can observe increased prevalence of abnormalities in carbohydrate and lipid metabolism, muscle atrophy and relative adiposity that increases the risk of coronary heart disease [21].

Individuals with SCI rely mostly on their upper body musculature for athletic activity. We have observed fundamental differences in the physiological adaptation between upper body and lower body exercise. Studies that investigate dynamic arm cranking and leg cycling in the same population show that maximal external power output (PO) and peak oxygen consumption (VO_2peak_) are lower during arm cranking [1]. Studies run by Pelliccia et al. showed the same tendency with VO_2_ peak median—27.1 mL/min/kg in athletes with SCI compared to 38.5 mL/min/kg in those with NSCI [8]. Such changes have been observed in male athletes in particular.

The studies performed by Baumgart et al. compare peak oxygen uptake and exercise efficiency between upper body poling and arm crank ergometry in able-bodied and paraplegic sportsmen [22]. In their research, the paraplegic participants included a wheelchair curler, an ice sledge hockey player, two hand-cyclists and two recreationally trained participants. The able-body group consisted of sub-elite cross-country skiers. To minimise the differences, the upper body was fixed, and the legs were supported and fixed in both groups. The peak oxygen uptake—VO_2peak_—was 24% lower in paraplegic athletes compared to able-bodied participants. Moreover, the smaller active muscle mass involved during arm work limits oxidative capacity and leads to an early onset of muscle fatigue. There was no variance in the given peak power output between paraplegic and able-bodied participants, indicating equal efficiency in both groups [22]. We also observed lower cardiac stroke volume in SCI athletes, probably due to the absence of skeletal muscle venous pump in the inactive legs during upper body exercise [1,8].

In association with the type of disability, we can observe major differences in left ventricular dimensions and masses [8]. Athletes with SCI have smaller left ventricular end diastolic dimension comparison with athletes with NSCI. Furthermore, the resting heart rate was lower in athletes with non-spinal cord injuries than sportsmen with spinal cord injuries. No differences were observed in relation to age, body size or systolic and diastolic blood pressure between NSCI and SCI athletes [8,23]. As mentioned, based on the type of discipline, we can divide athletes into those engaged in endurance sports and those involved in non-endurance disciplines. With regard to the type of sports, athletes in endurance sports had larger left ventricular cavities—median 52.0 mm vs. 49.0, greater wall thicknesses—with a median value of 10 mm vs. 9 mm and greater LV mass compared with athletes in non-endurance sports [8]. The smaller dimension is the result of several determining factors. One is the disruption of the descending autonomic pathways, which causes deviation in various organ systems, especially cardiovascular function, and results in the loss of supraspinal regulation of sympathetic activity [24,25,26,27,28]. The alternation in the left cavity volume and low increase in cardiac output occurs secondary to a SCI-induced reduction in blood pressure, total blood volume and reduced volume return [27]. According to Pelliccia et al., in individuals with SCI, we can observe a reduction in the leg’s muscle pump during exercise, which causes a relative entrapment of peripheral venous blood below the level of the spinal cord lesion, resulting in a reduced preload [8].

As is the case for able-bodied athletes, the type of sport has been found to be a determinant for cardiac remodelling in Paralympic athletes [29,30]. In particular, the endurance disciplines such as hand-cycling, cycling, rowing and cross-country skiing were associated with significant cardiac remodelling, especially in athletes with SCI, which was also previously mentioned [31].

### 3.2. Heart Rate and Electrocardiographic Features in Paralympian Group

One of the essential parts of physical examination is the 12-lead electrocardiogram. The prevalence of the ECG varies in paralympic athletes worldwide. According to Pelliccia, the electrocardiographic abnormalities that were not associated with training and were potentially an expression of cardiac disease were more frequently observed among female athletes and athletes with SCI [8]. The proportion of abnormal ECGs in the overall Paralympic athletes was not very different from what has been previously reported in athletes who are able-bodied, but most of these abnormalities were seen among athletes with SCI [32,33]. According to Sheikh et al., 1.25% of athletes who underwent ECG and echocardiography examination were diagnosed with cardiac disorders such as hypertrophic cardiomyopathy, Wolff-Parkinson-White syndrome, long-QT syndrome, Brugada syndrome and anomalous coronary artery origin [34]. Studies of Papadakis et al. showed no significant difference in the overall prevalence of T-wave inversions between adolescent athletes and the control group. Moreover, most individuals with T-wave inversions present a structurally normal heart with no evidence of an intra-cardiac shunt. This can lead to the conclusion that T-wave inversion is one of the cardiovascular adaptations of highly trained adult athletes [35].

The Pelliccia study from 2016 shows that systemic hypertension was present in 4% of disabled participants and 25 athletes (9%) suffered from supraventricular or ventricular tachyarrhythmias, especially atrial flutters and a paroxysmal burst of atrial fibrillation [36]. A comparison of two large-scale studies of non-disabled athletes showed that in 1.15% and 1.25%, respectively, electric diseases have been found [8,34]. Abnormal ECG findings which were not associated with training were more frequent in SCI athletes than in NSCI (11.8% vs. 4.2%) [8]. The most common findings were T-wave inversion (5.9%) and premature beats (more than two (1.5%)). Borderline ECG findings were seen in seven individuals (2.8%) and included right-axis deviation, isolated left-axis deviation and complete right bundle branch block [8]. There was no difference in the distribution of these issues in association with the type of disability or sport.

Moreover, in studies from 2021, the authors found that other ECG changes typically present in athletes as a physiologic consequence of exercise training, including increased R-wave or S-wave voltages, ST-segment elevation and incomplete right bundle branch block, were seen in about one-third of Paralympic athletes [8]. A similar tendency was observed among Brazilian elite disabled athletes. Signs of athlete’s heart, such as sinus bradycardia/arrhythmia, T-wave juvenile pattern and ventricular hypertrophy, were found in 55% of electrocardiograms [37]. It has been suggested that the prevalence of abnormal ECGs, suggestive of cardiac diseases, in participants with SCI may be the result of the greater prevalence of cardiovascular risk factors and cardiac or systemic comorbidities in these participants [8,36]. The most frequent ECG alterations were T-wave inversions. The athletes were not diagnosed with any cardiac disease; however, they were placed under greater supervision.

In the widely commented studies by Pelliccia, in which ergometers suitable for the specific disability were applied, the resting heart rate was higher in male athletes with SCI, with median 68 (61–75) bpm than male athletes with NSCI, and this was believed to be the consequence of the smaller LV cavity (and stroke volume) [8]. Additionally, blood pressure was comparable among the two groups in both sexes. Resting heart rates were lower in male athletes in endurance sports than non-endurance sports (the median was 60–75 bpm vs. 55 (51–63) bpm) [8]. When considered with regard to disability, Pelliccia et al. describe a slightly higher heart rate in athletes using wheelchairs than in others (70 ± 11 vs. 60 ± 10 and 66 ± 13), and blood pressure was moderately lower in athletes with visual impairments [36].

Another study that analysed the hemodynamic responses in two groups of conventional powerlifting (CP) and Paralympic powerlifting (PP) athletes shows the significant differences between systolic blood pressure (SBP) before and after powerlifting exercise [38] (Aidar, Paz i Gama, 2021). In both groups, an increased in SBP after training was noticed, with higher values for CP sportsmen. The Paralympic individuals had lower SBP afterwards and 60 min later, presenting a hypotensive effect even after 50 min. This effect is indicative of cardiovascular behaviour and can be induced by various mechanisms. It might be explained by the cardiac output, systolic volume and occlusion of vessels and arteries in addition to peripheral vascular resistance during exercise [38,39] (Paz i inni, 2020).

### 3.3. Cardiovascular Abnormalities in Paralympians

One of the most common arrhythmias, atrial fibrillation (AF), has a U-shaped dose–response in relation to endurance exercise [9]. On the one hand, study from 2008 showed that low-to-moderate-intensity exercise has been related to a decreased risk of atrial fibrillation [40]. On the contrary, a higher prevalence of AF was seen in individuals that performed endurance exercise with higher frequency—more than 4 times/week or for longer that 5 h/per week [41,42,43]. A meta-analysis from 2009 showed that endurance athletes are 5.3 times more likely to develop atrial fibrillation than the control population [44]. However, the authors indicate some controversies and suggest interpreting the results cautiously for several reasons, such as: the samples of the studies were small and controls were not appropriately age-matched in all studies; the results could be associated with some bias attributed to the variation in the level of endurance practiced by the different types of athletes; and, finally, most studies were performed in men, whereas the risk for women has not been well investigated. In conclusion, it should be stated that more research studies in this field are needed.

The majority of paralympic and Olympic athletes present a normal cardiovascular status, although a small percentage of them may present cardiovascular abnormalities. The systemic investigation of athletes evaluated in a 10-year period, from the Olympic Games in Athens 2004 to Sochi 2014, shows that cardiovascular (CV) abnormalities occurred in 92 (3.9%) Olympic athletes, and 11.9% of the total number of participants showed ECG or echocardiographic changes [2]. Considering the subset of Paralympic athletes, another study by Pelliccia et al. shows that CV abnormalities were found in 33 (12%) athletes and 9% were diagnosed with cardiovascular structural disorder [32]. Both groups of participants were examined with history, physical examination, 12-lead and exercise ECG and echocardiography [2,36]. In that study, the paralympic athletes participated in sports disciplines such as table tennis, equestrian, shooting, archery, curling, sailing, javelin, shot putting, short-distance running, alpine skiing, judo, basketball, ice-sledge hockey, tennis, fencing, rowing, swimming, cycling, hand-cycling, long-distance and marathon running and cross-country skiing [36].

In the aforementioned studies, the most common abnormalities identified in paralympic athletes were the arrhythmogenic cardiomyopathies (n = 3), aortic root dilatation (n = 3), mitral valve prolapse (n = 4), bicuspid aortic valve (n = 3) and systemic hypertension (n = 11) [36]. The data are presented in Table 1.

One of these abnormalities, dilated cardiomyopathy, was diagnosed in a visually impaired skier. Hypertrophic cardiomyopathy was identified in a paraplegic yachter. Furthermore ventricular (polymorphic, couplets or non-sustained ventricular tachycardia) or supraventricular tachyarrhythmias (atrial flutter, paroxysmal atrial fibrillation or STV) were found in nine others [36]. Moreover, in 3 athletes, the echocardiography study discovered isolated dilatation of the aortic root (>40 mm) and 25 athletes were recorded with supraventricular or ventricular tachyarrhythmias. Among the mentioned arrhythmias, atrial flutter was found in a cyclist and a paroxysmal burst of atrial fibrillation in a swimmer. Both athletes had reported previous palpitations [36]. The sledge and rower hockey players were diagnosed with short run of supraventricular tachyarrhythmias. Five other athletes presented ventricular ectopic beats (VEBs) with couplets and a short run of non-sustained ventricular tachycardia [36].

To compare, among able-bodied athletes, the most common CV findings were valvular and congenital disease abnormalities that included mild elongation of the mitral valves without evidence of prolapse (n = 25) and mild asymmetric tricuspid aortic valve without raphe (n = 6). Among athletes, we observed congenital changes (7%) such as: atrial septal aneurysm, patent foramen ovale and corrected atrial septal defects [2]. The summary of pathophysiological changes is presented in Table 2.

## 4. Conclusions

It seems that a group of paralympic athletes, especially a group with spinal cord injury, are more prone to develop arrhythmias, arterial hypertension and hyperlipidaemia, including atrial fibrillation and atrial flutter, and this group may potentially have a higher risk of cardiovascular mortality. In ECGs of the disabled athletes with SCI, we more often find changes typically established as consequences of exercise training, such as T-wave inversions. The potential differences in cardiovascular status in the disabled athletes may depend not only on the class of impairment, but also on the sports discipline and environmental conditions, which makes the analysis relatively complex. Due to the relatively few data available and the existing discrepancies in this area, further research is necessary.

## 5. Future Research Directions

Studies on the frequency of abnormalities of the cardiovascular system in Paralympic athletes indicate the need to intensify preventive cardiology care for this group of athletes. Activities that may be proposed in this area may include more frequent analysis of ECG, application of 24 h ECG Holter monitoring and echocardiography, and regular contact with a cardiologist; for example, every 1–2 years. An orthostatic tolerance test with blood pressure measurement can be used to predict cardiovascular capacity during competitions and establish cardiovascular limitations within each discipline, taking the altered autonomic control into account. Complexity related to structural and anatomic differences in Paralympic participants requires specific strategies for managing training and competition load, prevention of dehydration and heat illness, and regular periodic health assessments.

## 6. Limitations of the Study

The main limitation of the study is still the scarce number of research studies including meta-analyses for Paralympic athletes. As commented in the article, in several original studies, the samples of the participants were relatively small and controls were not always appropriately age-matched, or there were other confounding factors. Another of the controversies is the insufficient number of sportswomen in comparison with sportsmen taking part in the study. Finally, some authors extrapolated the results from the general group of athletes into the Paralympic group, which may not fully reflect the problem, even if they describe similar disciplines from endurance or non-endurance group.

## Figures and Tables

**Table 1:** Prevalence of cardiovascular abnormalities in Paralympic athletes.

Total cardiovascular abnormalities	12%
Arrhythmogenic cardiomyopathies	1.12%
Aortic root dilation	1.12%
Valvular diseases	2.62%
Systemic hypertension	4%
Ventricular or supraventricular tachyarrhythmias (atrial flutter, paroxysmal atrial fibrillation or STV)	3.34%

Adapted from [8] Pelliccia, A.; Quattrini, F.; Cavarretta, E. Physiologic and Clinical Features of the Paralympic Athlete’s Heart. *JAMA Cardiol*. **2021**, *6*, 30–39.

**Table 2:** Summary of studies on main pathophysiological changes in cardiovascular system in Paralympic athletes.

Pathophysiological Changes	Probable Patomechanism
Limited maximal heart rate [1] Lower maximal external power output [1,8] and peak oxygen consumption (VO_2peak_) [1,2,8,19] Smaller left ventricular and end-diastolic dimension [8] Smaller LV mass index. [8] Hypertrophic and arrhythmogenic cardiomyopathy [32,34,35,37] Supraventricular and ventricular tachyarrhythmias [8,9,30,34] Cardiovascular abnormalities such as: aortic root dilation and valvular diseases [34] Systemic hypertension [34] Higher risk of hypothermia [10,11,12] Heat-stress-related symptoms and exertional heat illness [12,13] Post-exercise hypotensive effect [38,39]	Lack of sympathetic drive to the heart [1,45] Altered autonomic control [14,19,24,45] Smaller active muscle mass involved during exercise [1,11,14] SCI-induced reduction of blood pressure and volume return [20,22,23,25] Reduced thermoregulatory ability [11,13] and disruption of autonomic nervous system [11,20] Reduction in skeletal mass, increased metabolic heat production [11] Peripheral vascular resistance [38,39]

## Data Availability

Data sharing not applicable.

## Supplementary Materials

The following supporting information can be downloaded at: https://www.mdpi.com/article/10.3390/healthcare11071027/s1, Table S1. Cerebral Palsy International Sport and Recreation Association–CP–ISRA Classes; Table S2. Paralympic classes for vision impairment; Table S3. Classification Systems used in Paralympic Summer; Table S4. Classification Systems used in Paralympic Winter Sports.
