# Supplementary material for: The Prevalence of Cardiovascular Diseases in Paralympic Athletes
Source: Healthcare (Basel). 2023 Apr 4;11(7):1027. doi: 10.3390/healthcare11071027 (PMC10094457; doi:10.3390/healthcare11071027)
Supplement: Supplementary file 1 [file healthcare-11-01027-s001.zip › healthcare-2271059-supplementary.pdf]

Supplementary Materials

Table S1. Cerebral Palsy International Sport and Recreation Association – CP – ISRA Classes.

CP – ISRA Classes	
CP1, CP2, CP3, CP4	The classes describe athletes using a wheelchair during competition
CP5, CP6, CP7, CP8	The classes describe athletes who don't use a wheelchair during competition

Adapted from: J. Buckley; "Understanding Classification" A guide to the Classification System used in Paralympic Sports, 2011.

Table S2. Paralympic classes for vision impairment.

Paralympic classes for vision impairment	
B1	No light perception at all on either eye or may have some light perception but an inability to recognise the shape of a hand at any distance
B2	Ability to recognise the shape of a hand and the ability to perceive clearly will be up to 2/60. Visual field less than 5 degrees
B3	Ability to recognise shape of a hand and the ability to perceive clearly will be above 2/60 and up to 6/60. Visual field between 5 and 20 degrees

Adapted from: J. Buckley; "Understanding Classification" A guide to the Classification System used in Paralympic Sports, 2011.

Table S3. Classification Systems used in Paralympic Summer.

	**ARCHERY**
Open	Both standing athletes and those in a wheelchair, archers position themselves at a 90 - degree angle to the target and may use body support
W1	Archers may use either bow limited to 45lbs in draw weight and without magnifying sights
	**ATHLETICS**
	**Track and Jump**
T11-13	Vision impairment
T20	Intellectual Disability
T35 - 38	Coordination impairments – hypertonia, ataxia and athetosis
T40 - 41	Short stature
T42-44	Lower limb competing without prosthesis affected by limb deficiency, leg length difference, impaired muscle power or impaired passive range of movement)
T45-47	Upper limb/s affected by limb deficiency, impaired muscle power or impaired passive range of movement)
T61-64	Upper limb/s competing with prothesis affected by limb deficiency and leg length difference
	**Wheelchair racing**
T32-34	Coordination impairments – hypertonia, ataxia and athetosis
T51-54	Limb deficiency, leg length difference impaired muscle power or impaired passive range of movement
	**PARA BADMINTON**

WH1	Wheelchair/ severe impairment
WH2	Wheelchair/ minor impairment
SL3	Standing/ lower limb impairment/ severe
SL4	Standing/ lower limb impairment/ minor
SU5	Standing/ upper limb impairment
SH6	Standing/ short stature
**BOCCIA**	
BC1	Cerebral Palsy: CP1 + CP2 – who push the ball with the foot
BC2	Only CP2 – no assistance permitted
BC3	Severe CP1 with use of an assistive device to propel the ball
BC4	Non CP players with a severe locomotor dysfunction of all four extremities
**CANOE / KAYAKING**	
KL1/VL1	Athletes who propel with their arms only
KL2/ VL2	Athletes who propel themselves with their arms and trunk
KL3/VL3	Athletes with full function of their arms and trunk, and partial function in the legs.
**CYCLING – Para-cycling**	
Classes H1- H5	Classes for handcycling, athletes with lower numbers indicating restrictions in both upper and lower limbs and higher number indicating restrictions in lower limbs only.
Classes T1 – T2	Neurological impairments. T1 – athletes are only able to use a tricycle. T2 – more ability than class T1, includes incomplete polio, spinal cord lesions and cerebral palsy.
Classes C1 - C5	Athletes who can use a standard bicycle, with cerebral palsy, and mild to severe impairments.
Tandem Class B	Blind and visually impaired
**EQUESTRIAN – Para-equestrian**	
Grade 1	Athletes are mainly wheelchair users with impairment in their trunk or poor trunk balance and limitation or loss of function in all four limbs.
Grade 2	Athletes are mainly wheelchair users or those with severe movement disabilities.
Grade 3	Athletes are mainly able to walk without support.
Grade 4	Athletes have a disability in only one or two extremities or some visual impairment
**FOOTBALL**	
7 a side	Athletes with classes CP5, CP6, CP7 and CP8
5 a side	Athletes with the class B1, B2, B3 are eligible to compete
**GOALBALL**	
B1, B2, B3	
The athletes compete together in an open event, having their eyes covered. To be eligible, athletes have less than 10 per cent visual acuity remaining or a visual field restricted to 40 degrees diameter.	
**JUDO**	
B1, B2, B3	
The athletes compete together being placed in the same weight divisions that operate in Olympic judo.	
**POWERLIFTING**	
All eligible athletes are divided by gender and in weight categories.	
**ROWING – Adaptive Rowing**	
PR1	Rowers with minimal or no trunk function who primarily propel the boat through arm and shoulder function. Usually, the athletes require to be strapped to the boat/seat.
PR2	Rowers that have functional use of arm and trunk but with some weakness/absence of leg function to slide the seat
PR3	Rowers with residual function in the legs which allows them to slide the seat. Also, athletes with vision impairment.
**SHOOTING**	
SH1	Pistol and Rifle competitors that do not require a shooting stand. Athletes with upper and/or lower limb impairment.
SH2	Rifle competitors who have no ability to support the weight with their arms and therefore require a shooting stand
SG-S (Trap)	Athletes with poor balance and/or trunk stability with impairment in the lower limb(s), competing from wheelchair or seat in a standard seated position.
SG-L (Trap)	Athletes with good balance and trunk function with an impairment in the lower limb(s) but competing from a standing position.

SG-U (Trap)	Athletes with good balance and trunk function, competing from a standing position.
SH-VI (Rifle)	Athletes with a vision impairment.
**SITTING VOLLEYBALL**	
2 sport classes depending on the severity and impact on the core functions – VS1 and VS2 (less impaired).	
**SWIMMING**	
Classes 1-10	Swimmers with a physical disability
Classes 11-13	Swimmers with a visual disability
Classes 14	Swimmers with an intellectual disability
**TABLE TENNIS**	
TT1 - TT5	Sitting classes including players with impairment with all four limbs and normal arm and body movements
TT6 – TT10	Standing classes including players with combination of disability in the playing arms and legs or severe disability in one or both legs
TT11	Intellectually disabled athletes
**PARA TAEKWONDO**	
K43	Athletes with bilateral amputation below the elbow, or equivalent loss of function in both upper limbs.
K44	Athletes with unilateral arm amputation or loss of toes which impact the ability to lift the heel properly
**TRIATHLON**	
PTWC 1-2	Athletes with limitations in lower (PTWC2) and upper limbs (PTWC1), using handcycle for the cycling and racing chair for the running segment.
PT 2-5	Athletes with severe leg impairment including above knee amputees. Athletes may use crutches or prosthesis if required.
PTVI 1-3	Athletes with vision impairment.
**WHEELCHAIR BASKETBALL**	
1 point player to 4.5-point player	Players must have an objective and measurable permanent physical disability in their lower limbs which prevent them from running.
**WHEELCHAIR FENCING**	
A Category	Fencers have good sitting balance of the trunk without support of the legs or with legs support.
B Category	Fencers have an impairment that impacts their trunk or their fencing arm.
**WHEELCHAIR RUGBY**	
0.5 – 3.5	Athletes are grouped withing a point system from 0,5 points describing the most limited functional ability, up to 3,5 points describing the highest level of functional ability.
**WHEELCHAIR TENNIS**	
Open Class	Athletes with lower limb(s) disability, but with normal arm function
Quad Class	Athletes with additional restrictions in the playing arm.

Adapted from: https://www.paralympic.org/sports.

Table S4. Classification Systems used in Paralympic Winter Sports.

**PARA ALPINE SKIING**	
LW 1-4	Standing skiers with lower limb(s) impairments.
LW 5-8	Standing skiers with upper limb(s) impairments.
LW9	Skiers with combined upper and lower limb impairments
LW10-12	Sit-skiers with an impairment affecting their legs and compete in a seated position
B1-3	Athletes with vision impairment
**PARA SNOWBOARD**	
SB-LL1 – LL2	Athletes with an impairment in one or both legs
SB-UL	Athletes with an impairment in one or both arms
**PARA ICE HOCKEY**	
There is one sport class, and all athletes have an impairment in the lower part of their body.	

Adapted from: https://www.paralympic.org/sports
